# Comprehensive Flow Cytometry Analysis of PEI-Based Transfections for Virus-Like Particle Production

**DOI:** 10.34133/2020/1387402

**Published:** 2020-03-13

**Authors:** Daniel J. Blackstock, Alvenne Goh, Shamitha Shetty, Giulia Fabozzi, Rong Yang, Vera B. Ivleva, Richard Schwartz, Joseph Horwitz

**Affiliations:** Vaccine Production Program Lab, Vaccine Research Center, NIAID, NIH, Gaithersburg, MD, USA

## Abstract

The generation of stable clones for biomolecule production is a common but lengthy and labor-intensive process. For complex molecules, such as viruses or virus-like particles (VLPs), the timeline becomes even more cumbersome. Thus, in the early stages of development, transient production methods serve as a reasonable alternative to stable clone construction. In this work, an investigation of a polyethylenimine- (PEI-) based transfection method for the transient production of Chikungunya (Chik) VLPs, a vaccine candidate molecule, was undertaken. This effort focuses on tracking cell population responses during transfection, understanding how process changes affect these responses, and monitoring patterns in cell performance over the culture duration. Plasmid labeling and VLP staining were employed to comprehensively track cells via flow cytometry and to draw correlations between plasmid DNA (pDNA) uptake and the resulting VLP expression. The method detected high transfection efficiency (≥97%) in all samples tested and demonstrated the capability to track kinetics of plasmid-cell binding. With varied transfection cell concentrations, the pDNA binding kinetics are altered and saturation binding is observed in the lowest cell concentration sample tested in less than 3 hours of incubation. Interestingly, in all samples, the flow cytometry analysis of relative pDNA amount versus VLP expression staining showed that cells which contained fewer pDNA complexes resulted in the highest levels of VLP stain. Finally, to determine the potential breadth of our observations, we compared daily expression patterns of ChikVLP with a reporter, monomeric GFP molecule. The similarities detected suggest the interpretations presented here to likely be more broadly informative and applicable to PEI-based transient production of additional biological products as well.

## 1. Introduction

Transient transfections comprise the introduction of foreign nucleic acids into a host cell for the temporary expression of a gene of interest. Unlike stably transfected genes, transient transfections do not require the integration of DNA into the host chromosome [1]. Stable cell lines require a time- and labor-intensive selection and screening process to fulfill. Therefore, in the early stages of clinical drug development, transient transfections serve as a useful option for fulfilling and accelerating protein production needs [2–4]. Transfections can be performed using a variety of methods but can be classified within one of the following three classes: biological, chemical, or physical [5, 6]. Biological methods involve the use of viruses to introduce genetic material, also known as transduction; these are generally highly efficient, but disadvantages include lab hazards and mutagenesis [7]. Physical methods include those that physically alter the cells to temporarily compromise the cell membrane for translocation of nucleic acids inside the cells. Physical-based methods comprise the most recent developments for transfection and include direct microinjection, biolistic particle delivery, electroporation, and laser-based transfection. These methods are also reported to be efficient but require specialized equipment [8]. Finally, chemical methods involve the introduction of chemicals which form electrostatic interactions with nucleic acids and the anionic cell surface to result in endocytotic uptake of the nucleic acid/chemical complexes. Common chemical methods include the use of cationic polymers, calcium phosphate, cationic lipids, and cationic amino acids. These methods are generally reported to be efficient, but may result in chemical toxicity to the cells [9]. Our work here focuses on investigating a chemical-based method, specifically polyethyleneimine (PEI), for the transient production of the vaccine candidate Chikungunya virus-like particles [10]. PEI-based transfection has been used for many years and shown to be a viable option for transient systems due to low cost and high transfection efficiency [11]. Additionally, the potential scalability of PEI-based transfection has been demonstrated [12, 13], making it an advantageous option for the production of large quantities of biological reagents. When using PEI, the PEI to plasmid DNA ratio has been reported to have significant effects on plasmid delivery and expression. Additionally, literature suggests that transient expression works best when the cells are in the exponential phase of cell growth [14].

Chikungunya virus (ChikV) is an alphavirus (of the *Togaviridae* family) most commonly transmitted by the *Aedes aegypti* and *Aedes albopictus* mosquitoes. ChikV is a public health problem in tropical areas of Africa and Asia and a threat to temperate areas colonized by *Aedes* mosquitoes, such as Europe and the Americas [15]. ChikV is an enveloped positive-strand RNA virus, composed of 12,000 nucleotides encoding four nonstructural and five structural polyproteins. The structural proteins are translated from a subgenomic 26S mRNA as a single polyprotein and subsequently processed by capsid autoproteinase and signalases into the five products: capsid, E3, E2, 6k, and E1 [16]. The virion consists of 240 copies of capsid protein and a host-derived lipid envelope embedded with 240 heterodimers of E1-E2. The E1-E2 heterodimers form 80 trimeric viral spikes on the surface of mature virions, and these epitopes induce neutralizing antibody responses to infection or vaccination. Noninfectious Chikungunya virus-like particles (ChikVLPs) which lack the nonstructural proteins have shown strong immune response in nonhuman primates [10] and humans [17].

Although PEI transfections have been widely applied, variable results still occur due to a lack of knowledge in understanding many of the molecular activities of the transfection process. If transient systems are to be applied for dependable clinical material generation, then more knowledge is needed for reproducibility and outcome predictability. There are minimal studies which directly assess the distribution of PEI-pDNA uptake across the cell population and monitor the resulting relative expression levels within the population for understanding transfection. In particular, breaking down the bulk cell population into subpopulations rather than focusing on the overall (mean) outcome allows us to determine how the various subpopulations are likely contributing to the end result. From this, we can gain a deeper interpretation of the results and ultimately a better understanding of how to reproducibly generate successful transient productions. Thus, in this work, to further elucidate the transfection process, we use fluorescent labeling technologies and flow cytometry [18] to thoroughly track cell responses. We focus on the kinetics of transfection, cell surface protein staining, and protein expression profiles to help derive correlations between plasmid delivery and the resulting expression levels.

## 2. Results

### 2.1. PEI Transfection and Flow Cytometry Strategy

Transfection conditions previously applied to the transient production of influenza vaccine candidates HA-ferritin [19] and H1-ss-nanoparticle [12] were applied here. The transfection procedure included collecting cells during the exponential growth phase and introducing a cell concentration step to reach 20*e*6 cells/mL [2] prior to transfection, then adjusting the culture with fresh media to reach a target concentration of 10*e*6 cells/mL for protein expression. A pDNA : PEI ratio of 1 : 2 (20 *μ*g/mL : 40 *μ*g/mL) was used for all transfection samples.

Although transfection working conditions could be inferred by simple titer analysis, understanding the percentage of transfected cells and the expression response across the population requires a different approach. Characterization of cell expression dynamics is key to establish and determine if transfection conditions are being maximized. In order to gain this enhanced understanding, flow cytometry [18] analysis was implemented. The characterization included labeling ChikVLP plasmid, ChikVLP-expressing cells, and dead cells. The plasmid was labeled with Cy5 molecules (Mirus Bio) prior to transfection and retained expression competence. The plasmid label served to not only investigate the percentage of cells receiving the plasmid but also draw correlations between relative plasmid levels per cell and the resulting expression profiles. A m242 monoclonal antibody [20, 21] specific to the ChikVLP was labeled with Alexa488 (ThermoFisher) for cell surface staining of live cells to determine VLP-expressing cells and the relative expression level. The dead cells were stained with propidium iodide for complete exclusion from the analysis. The gating strategy for all analyzed samples is captured in [Supplementary-material supplementary-material-1].

### 2.2. Kinetics and Efficiency of PEI-Based Transfections

**Figure 1:**
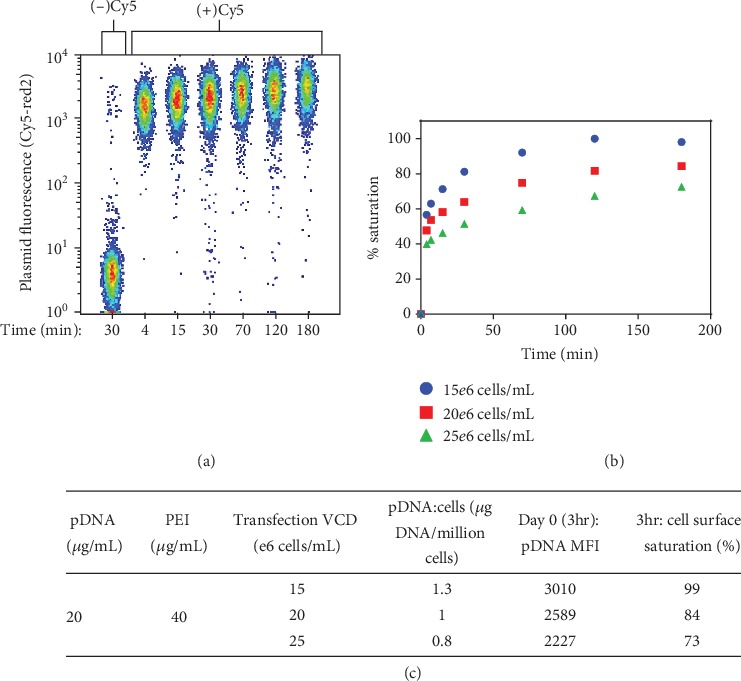
Flow cytometry analysis of the kinetics of pDNA : PEI complex binding to cells. (a) The 20*e*6 cells/mL transfection density sample with (-) Cy5 population included as a control transfection with pDNA lacking the Cy5 label. (b) PEI-pDNA complex-cell surface binding kinetics for transfection cell densities of 15*e*6, 20*e*6, and 25*e*6 cells/mL. The % saturation reflects the estimated percentage of binding sites on the cell surface occupied by PEI-pDNA complexes. The plasmid median fluorescence intensity (MFI) value achieved upon plateaued signal was used to determine the % saturation value. (c) Table summarizing transfection conditions for each sample and the corresponding measurements of pDNA MFI and cell surface saturation levels at 3 hrs posttransfection.

Captured using flow cytometry, Figure 1(a) shows the kinetics of pDNA complex-cell binding and the cell population receiving the plasmid. The baseline fluorescence was established by setting the background fluorescence in 1-log using a control transfection sample, in which cells were transfected with a plasmid lacking the Cy5 label. As displayed in Figure 1(a), the entire cell population binds with the plasmid complex within just 4 minutes or less after component addition. In addition to the complex-cell binding kinetics, the PEI-pDNA complexing rate was analyzed using an agarose gel and DNase treatment procedure as described in Materials and Methods. As shown in [Supplementary-material supplementary-material-1], after the two components are mixed, PEI-pDNA complexes form and protect the pDNA from degradation by the DNase added. Rapid complexing was observed, as within 4 minutes or less of mixing, the PEI has protected the pDNA from degradation. This PEI-pDNA complexing data was in line with the complex-cell binding results captured in Figure 1(a).

We hypothesized that flow cytometry would be effective in identifying small changes within the cell population dynamics in respect to plasmid delivery and VLP expression. Thus, to understand how potential transfection procedure variations could affect the experimental outcome, we varied the target 20*e*6 cells/mL transfection density by ±25%. To normalize the resulting growth and titer yield, each transfection sample was adjusted to 10*e*6 cells/mL with fresh medium posttransfection, as described in Materials and Methods. At all transfection densities tested, it was observed that approximately 97% of the cells were bound with the complex ([Supplementary-material supplementary-material-1]). However, the % saturation of cell surface binding sites, shown in Figure 1(b), indicates that relatively small changes in cell density and transfection time can play a substantial role in determining the amount of complexes associating with each cell. For instance, at 30 min binding time, approximately 81% of surface binding sites were occupied for the 15*e*6 cells/mL transfection density condition, 64% for 20*e*6 cells/mL, and 51% for 25*e*6 cells/mL. These observed differences in binding can be attributed to the effective changes in the pDNA complex : cell ratios, summarized in Figure 1(c), where higher cell densities result in fewer complexes bound per cell when pDNA/PEI concentrations are kept constant. As noted in Figure 1(b), the 15*e*6 cells/mL transfection sample had the highest pDNA complex : cell ratio, 1.3 *μ*g : million cells, and the cell surface becomes saturated with the complex around 120 min posttransfection. However, the two other higher cell density samples do not even reach saturation binding within the 180 min transfection period. Based on previous reports, the PEI complex internalization occurs within 1-2 hrs or less posttransfection [22, 23], and daily flow cytometry tracking was performed to ensure that the cells contained plasmid throughout the culture.

**Figure 2:**
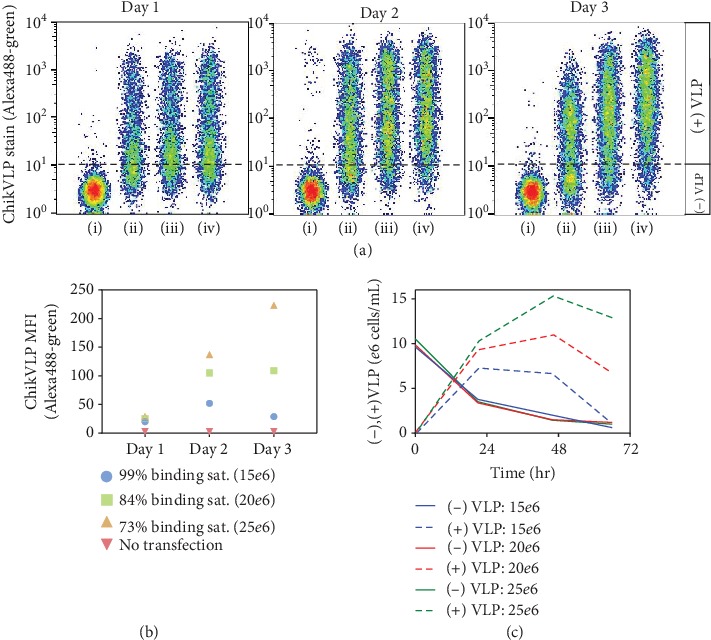
Flow cytometry analysis of PEI transfected cells stained with Alexa488 labeled ChikVLP mAb. (a) Samples at the different transfection cell concentrations were concatenated for day 1, day 2, and day 3 expression analysis. Samples are defined as follows: (i) nontransfected and transfected at (ii) 15*e*6 cells/mL, (iii) 20*e*6 cells/mL, and (iv) 25*e*6 cells/mL. (b) Plot of the daily VLP MFI for each transfection sample which is denoted as the level of pDNA complex-cell saturation binding measured at 3 hrs posttransfection. (c) The calculated (-) and (+) VLP cells for each sample during the first 72 hours of culture. The daily VCD and the percentage of VLP staining cells found in (a) were used to estimate the VLP-producing cells.

**Figure 3:**
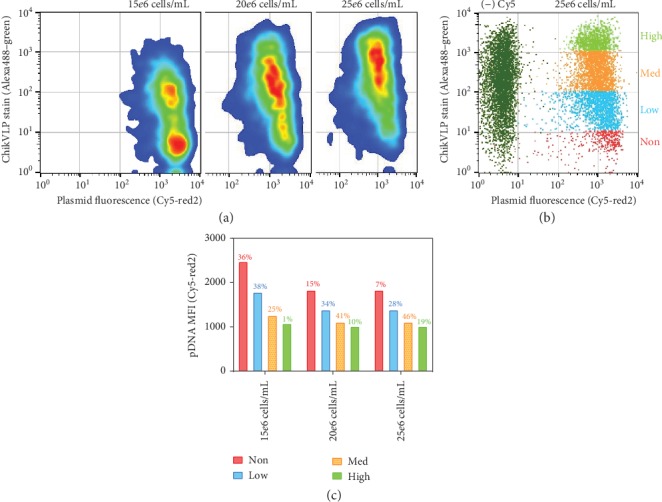
Analysis of transfections based on ChikVLP staining vs. plasmid fluorescence. (a) Day 3 flow cytometry cell population heat map for each sample tested. (b) Flow cytometry results of the 25*e*6 cells/mL sample grouped into different expression (high-, medium-, low-, and non-) staining fractions and plotted against the control (-) Cy5 transfection sample. (c) Plasmid MFI of each VLP staining fraction for each transfection cell density (bar graph). The percentages (%) listed above bars represent the cell population fraction within that staining classification.

An anti-E2 mAb 242 [20, 21] specific for the E2 protein domain of the VLP envelope was used to track the relative VLP expression levels (Figure 2(a)). Although, the anti-E2 mAb does not directly measure VLP particles, the E1-E2 protein domains accumulate at the cell surface and provide a relative way to measure transfection and expression efficacy of VLP production. During the formation of fully functional VLPs, the VLP capsid is coated with the E1/E2 domains upon VLP release. Thus, from here on, we term positive cell binding with the anti-E2 mAb as VLP stained or (+) VLP cells. We found that the initial day 1 expression profile was similar among all samples; however, by culture days 2 and 3, the differences in profiles between samples became more apparent. The daily VLP median fluorescence intensity (MFI) levels measured for each transfection density were plotted and referenced according to the initial plasmid complex-cell binding levels detected (Figure 2(b)). On day 2 posttransfection, the 15*e*6 cells/mL transfection condition (99% cell saturation) yields about 2-fold lower VLP stain compared to 20*e*6 cells/mL sample (84% cell saturation) and 2.6-fold lower compared to the 25*e*6 cells/mL (73% cell saturation). Then, on day 3, this discrepancy is further enhanced to 4-fold (84% saturated) and 8-fold (73% saturated) as shown in Figure 2(b). In Figure 2(c), based on the viable cell density (VCD) and percentage of (+) and (-) VLP cells, the daily amount of cells staining in each sample was calculated. Interestingly, regardless of the growth profiles, the numbers of (-) VLP cells were consistent across samples for any given day. This trend suggests that the nonproducing cells are becoming producing cells at the same rate for all samples, regardless of the amount of pDNA within the cell. However, the producing cells in the 15*e*6 cells/mL sample are dying more rapidly, thus yielding lower levels of (+) VLP cells. Utilizing the pDNA label, the VLP staining was analyzed for the relative pDNA level (Figure 3(a)). In all samples, the entire cell populations appear to contain plasmid; however, a fraction of cells, ~1*e*6 cells/mL (Figure 2(b)), still do not stain for VLP production by day 3. Similar observations were previously reported even though the pDNA was confirmed to have entered the cytoplasmic space and cell nucleus [23, 24].

### 2.3. Correlating Cellular Responses with Plasmid Delivery

Figure 3(a) shows an inverse staining trend where nonstaining cells actually indicate that a higher level of pDNA-PEI complex is present. To better gauge the trend, the cell population was grouped into non-, low-, medium-, and high-VLP staining fractions (Figure 3(b)), and the pDNA MFI for each fraction was graphed (Figure 3(c)) along with the cell population percentage (indicated above the bar) within those groups. In Figure 3(c), the 15*e*6 cells/mL sample, which received the most plasmid complex/cell upon transfection (Figure 1), shows the highest percentage of non-VLP cells (36%) and low-VLP cells (37%), whereas the 25*e*6 cells/mL sample received the least amount of plasmid complex/cell but produces a trend of the most cells staining in the mid-VLP (46%) and high-VLP (19%) cells. This trend is further emphasized, by the pDNA MFI for each staining fraction, as there is a trend across samples of around 2-fold less pDNA in the high-VLP staining cells compared with the nonstaining cells (Figure 3(c)).

**Figure 4:**
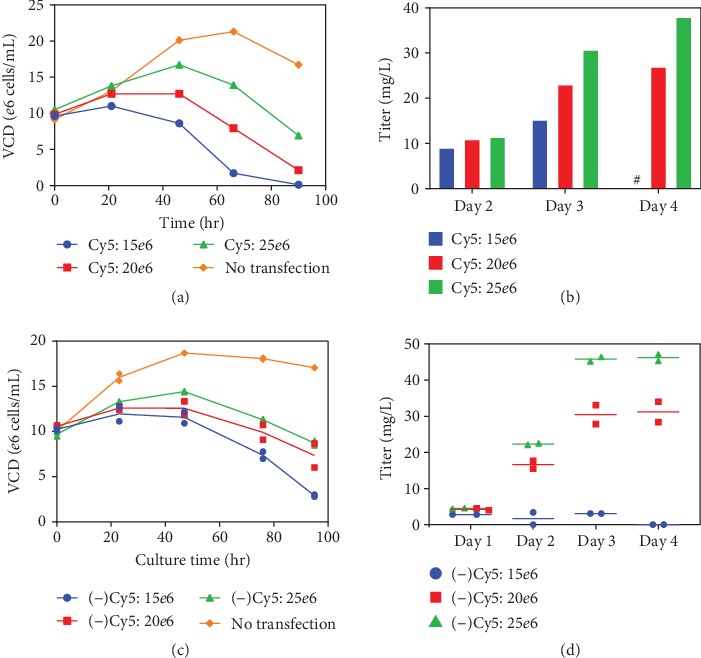
Cell growth and titer analysis of transfections performed with and without Cy5-labeled ChikVLP plasmid. Cell growth profiles for varied transfection density samples using Cy5-labeled (a) or nonlabeled (c) pDNA. VLP titers were measured daily for each transfection sample for the Cy5-labeled (b) or nonlabeled (d) pDNA transfections. The nonlabeled plasmid samples were run in duplicates and plotted as individual symbols with the duplicate averages illustrated by a horizontal line. ^#^Day 4 titer for the (+) Cy5-15*e*6 cells/mL sample was not reported due to associated assay error with complete cell lysis. Day 4 titer for the (-) Cy5-15*e*6 cells/mL was below the limit of quantitation and assumed to be zero.

Figure 4(a) shows the growth profiles for each sample posttransfection and reveals that the higher transfection density sample, 25*e*6 cells/mL, which contains the least plasmid/cell on average, demonstrates the best growth posttransfection and ultimately the best staining percentage for VLP production (Figure 2(a)). However, the inverse VLP stain-to-pDNA trend is observed across all samples, even when minimal growth is observed, as in the case of the 15*e*6 cells/mL transfection sample (Figure 4(a)). Thus, the trend also suggests that within each transfection sample, there is a healthier (lower) amount of PEI-pDNA complex received in some fractions of the cells (Figure 3(c)—the orange and lime green populations), and thus, those populations yield a better VLP expression response. The higher levels of PEI-pDNA complex could result in PEI toxicity effects, reduced growth, and inefficient processing and unpacking of the pDNA complex [25]. The observed trend highlights the importance of delivering a controlled amount of complex to achieve desirable growth and expression. The degradation in the plasmid fluorescence signal was tracked daily for all samples and reported in [Supplementary-material supplementary-material-1]. A similar degradation profile was noted in all samples with the most substantial loss within the first 24 hours (~35% loss). The signal loss is thought to be attributed to cytosolic release of pDNA, photobleaching, and/or dilution effects based on cell growth [26].

The day 2-4 titers are shown in Figure 4(b) and confirm that the 25*e*6 cells/mL transfection condition yields the best titer, reaching about 40 mg/L by day 4. It is noted that the Cy5 label likely causes hindrance between DNA polymerase binding to the plasmid DNA backbone, potentially reducing the overall level of transcription [27]. Regardless, since the same batch of Cy5-pDNA is used for all experiments, the observed trends should stand independent of the pDNA label. In order to confirm the trends observed, the transfection experiment was repeated in duplicates using nonlabeled ChikVLP plasmid under the exact same conditions described for the Cy5-pDNA. However, it is also important to note that these transfections were carried out using different growth medium lots, PEI lots, and supplemental feed lots. As shown in Figure 4(c), the growth profiles for the varied transfection cell densities resemble those observed in Figure 4(a), where the 25*e*6 cells/mL sample yields the best. Additionally, the titer trends (Figure 4(d)) are in line with those observed in Figure 4(b), such that the 25*e*6 cells/mL transfection condition demonstrates higher daily VLP titer by day 2 posttransfection and reaches an average titer of 45 mg/L on day 4. The 15*e*6 cells/mL sample yielded poorer cell health and growth and only reached a maximum titer of 5 mg/L ChikVLP, which was even lower than that observed for the labeled plasmid culture. The daily cell counts, viabilities, and cell-specific productivities for all Cy5 and (-) Cy5 samples are reported in [Supplementary-material supplementary-material-1]. Based on the compiled data, it is clear that when cells reach saturation binding of the pDNA complex, achieved by lowering transfection cell density (or increasing the pDNA complex : cell ratio) and/or extending transfection time, the culture is negatively affected. Ultimately, the analysis for the highest delivery condition showed reduced VLP staining, minimal cell growth, low cell viability, and low-VLP titers.

### 2.4. Comparing ChikVLP and GFP Expression Profiles

**Figure 5:**
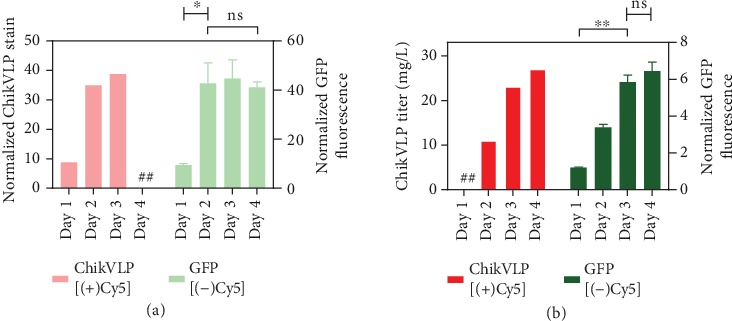
Daily expression trends for ChikVLP (Cy5-labeled pDNA) and GFP (nonlabeled pDNA) transfections. Transfections carried out at a cell density of 20*e*6 cells/mL are plotted for daily (a) VLP cell stain or cellular GFP fluorescence and supernatant measurements of (b) VLP titer or fluorescence. ^##^Data was not collected for these culture days. The ChikVLP stain and GFP fluorescence were normalized by dividing the measured signal by the daily background recorded for the nontransfected cell population, such that the background signal in each case is made equal to one. The culture media yielded a significant emission response at the desired wavelength which explains the lower fold increase in fluorescence detection when analyzing the culture supernatant. Results of a 1-way ANOVA for the GFP measurements are reported (ns = not significant, ^∗^*p* value < 0.05 or ^∗∗^*p* value < 0.01).

To determine the applicability of the observations beyond the complex multimeric VLP, the expression profile of a monomeric GFP molecule containing a secretion peptide tag and the same promoter elements of ChikVLP was investigated. Following the same procedure executed for the Cy5-labeled ChikVLP pDNA, transfection at the 20*e*6 cells/mL density was performed and monitored for GFP expression. The cells and the culture supernatant were analyzed daily for fluorescence emission to determine the relative expression levels over the course of a four-day culture. In Figure 5(a), daily VLP staining MFI via flow cytometry is compared to the GFP emission signal observed for 1.5*e*6 cells. The kinetics of expression appear to be very similar, where day 1 shows a low level of expression, but by day 2, maximum intracellular levels are reached. Further analysis of the culture supernatant (Figure 5(b)) shows that GFP molecules and VLPs are being secreted at a very similar rate where the secretion kinetics dramatically slow down by day 3 and relatively minor product gains are observed thereafter. Based on the day 3 flow cytometry results in Figure 3(a), this slow down appears not to be a result of plasmid loss or dilution as essentially the entire cell population contains plasmid but likely an effect of the culturing conditions. Additionally, it has been shown that an intensified culturing or supplementation method can extend the culture duration and ultimately achieve significantly higher final harvest titers [12]. Nonetheless, under the culturing conditions tested here, the expression trends agree for complex (VLP) and monomeric (GFP) molecules with and without Cy5 plasmid labels, so it appears that the flow cytometry insight and interpretations can likely be broadly applied to PEI-based production systems.

## 3. Discussion

In this work, flow cytometry is used to investigate cell transfection kinetics and VLP expression profiles throughout culture duration and determine how changes in transfection conditions can affect these outcomes. The approach helps to explain the anticipated cell responses and provides insight on trends observed at the molecular level. The rapid kinetics of PEI-pDNA complexing ([Supplementary-material supplementary-material-1]) and the complex-cell binding kinetics (Figure 1) were captured for a range of transfection cell concentrations. High transfection efficiency was noted in all samples tested; however, it was demonstrated that transfection cell concentrations differing by 25% can have substantial impacts on total cellular complex delivery levels. The variation of cell concentrations effectively alters the ratio of the pDNA complex to cells and results in different levels of complex binding and delivery to cells (Figure 1(b)). Additionally, the results indicate that high plasmid delivery or high transfection efficiency does not necessarily translate to successful expression levels. In particular, when using a pDNA concentration of 20 *μ*g/mL (with a 1 : 2 ratio of pDNA : PEI) and a transfection cell density of 15*e*6 cells/mL, the pDNA complex : cell ratio becomes 1.3 *μ*g pDNA complex/million cells and the cell surface binding reaction reaches saturation within approximately 2 hrs. Although this condition results in high levels of complex delivery, the subsequent cell growth and productivity yields are very poor (Figure 4). Alternatively, when the transfection pDNA complex : cell ratio is lowered to 0.8 *μ*g pDNA complex/million cells (in the case of 25*e*6 cells/mL), the binding level is reduced to 73% cell saturation at 3 hrs of transfection time (Figure 1(b)). At this reduced delivery level, the cell growth is improved, the (+) VLP staining cells are increased by more than 3-fold (Figure 2(b)), and the VLP yields are dramatically improved (Figure 4). Thus, the transfection cell concentrations, or pDNA complex : cell ratio, can be a point of manipulation to control the complex binding levels.

In conjunction with controlling the complex : cell ratio, the transfection time is another variable which can be managed to regulate the complex-cell binding levels. For example, to reach a desired cell binding level of approximately 70% saturation (Figure 1(b)), the binding kinetics data showed that a transfection time of 180 min was necessary for 25*e*6 cells/mL (0.8 *μ*g pDNA complex/million cells), 55 min for 20*e*6 cells/mL (1.0 *μ*g pDNA complex/million cells), and only 15 min for 15*e*6 cells/mL (1.3 *μ*g pDNA complex/million cells). This means that at high pDNA complex/cell ratios, the transfection process can potentially be shortened to just minutes while still delivering the desired pDNA complex concentration. Ultimately, this type of “transfection time”-based control approach may be advantageous to some production processes where the transfection cell density is bound to be variable.

This work focuses on the evaluation of a transfection process at higher transfection cell densities of 15-25*e*6 cells/mL, which is particularly useful for intensified cell culturing processes, where high cell densities and elevated titers are desired and achieved. However, since the cell responses are thought to be a function of complex delivery levels, the trends and outcomes observed here as a function of cell saturation binding are anticipated to apply to low cell density transfections as well. However, it is noted that transfections at low cell densities would require a reduction in the pDNA/PEI concentrations to ensure nonsaturation binding; therefore, a precomplexing step of pDNA-PEI may be considered, which was not performed in this work.

Future focus could include cell sorting to isolate the high-VLP stained cells and determine the absolute number of plasmids for quantification of ideal delivery levels to accomplish optimal cell performance. However, upon determining this number, achieving a very uniform delivery within only the range of high producers may be challenging due to the natural distribution of delivery observed with PEI transfection. Additional work could also involve looking at other means of transfections for the comparison of cell responses between methods. It would be interesting to note if similar observations are found with other chemical-, physical-, and viral-based methods and if the distribution of plasmid delivery can be more concisely controlled with a given method. Overall, the flow cytometry evaluation helps elucidate the cellular responses for PEI-based transfections for VLP expression, but it is believed that the observations can be broadly applied for the transient, PEI-based, production of other molecules as well.

## 4. Materials and Methods

### 4.1. Experimental Design

The experimental objectives sought to determine transfection efficiency, relative pDNA delivery levels, trends of cell expression at various levels of delivery, and daily changes within the system. Flow cytometry was employed to offer the advantages of live cell population analysis, by analyzing thousands of cells (20,000 events) for an accurate representation of behavior among the entire sample instead of focusing on responses from selected few cells. The experimental designs included variation of transfection cell concentrations to observe how manipulations in pDNA delivery may impact results. The labeled pDNA and PEI concentrations were held constant, and 3 samples were run at varied cell densities. A positive control (lacking the Cy5 pDNA label) at the center point transfection cell density and a negative control (identical processing, but no transfection) were performed for each method.

### 4.2. Cell Culture: Transfection, Expression, and Sampling

Serum-free, suspension-adapted HEK293 cells were subcultured in CDM4HEK293 (HyClone, GE, Utah, USA) media two times per week. The cells were grown in single-use shake flasks up to the 3 L scale, with settings of 25 mm shaking diameter, 130 rpm, 37°C, 80% humidity, and 6% CO_2_. For all transfection experiments, cells were gathered during the early exponential growth phase. The ChikVLP plasmid used in all transfection experiments was VRC8900 [10], carrying an open reading frame consisting of the Chikungunya capsid, E3, E2, 6k, and E1 under the CMV/R promoter and 8,159 bp in size. The GFP plasmid used was VRC3925 which carries an enhanced GFP (eGFP) gene sequence in-frame with a sequence coding for mPER HIV epitope. The GFP-mPER gene was also under the CMV/R promoter and is secreted for measurement in culture supernatant.

For PEI (linear 25 kDa, Polysciences Inc., Warrington, PA) transfection experiments, the cells were collected via centrifugation at 200 × g for 10 min, the supernatant was discarded, and the cells were resuspended in Freestyle 293 (ThermoFisher) media to reach cell concentrations of 15*e*6, 20*e*6, and 25*e*6 cells/mL and then distributed into separate 125 mL shake flasks. The with (+) Cy5 or without (-) Cy5 pDNA were added first to the culture at a 1 : 50 dilution to reach 20 *μ*g/mL and swirled to ensure proper mixing. The PEI was then added at a 1 : 50 dilution to a final concentration of 40 *μ*g/mL and immediately placed into the shaker. Three hours after PEI addition, the cells for each shake flask were spun down at 200 × g for 10 min, the supernatant was removed, and the cells were resuspended in CDM4HEK293 media to a target cell concentration of 10*e*6 cells/mL. At ~24 hours posttransfection (hpt), 4 mM valproic acid (VPA) was added to each flask, and at ~48 hpt, each flask was fed with 6 g/L glucose and 3 mM L-glutamine.

The same procedure described above was used for GFP transfection experiments and performed in duplicates; however, only the 20*e*6 cells/mL transfection density was tested. Additionally, following transfection in shake flasks, the cultures were transferred to an AMBR15 bioreactor instrument for culturing and the vessels were controlled at a 50% DO setting and a pH setpoint of 7.3+/-0.1.

### 4.3. VLP Quantitation

Daily samples were taken from each flask for cell counts (Cedex HiRes Analyzer, Roche, France) and VLP titers. All titer samples were syringe filtered using a 0.2 *μ*m filter prior to VLP quantitation. In order to overcome the complex matrix interference from the culture media and host cell proteins, a tandem chromatographic method combining ion-exchange (IEX) and size-exclusion (SEC) columns was applied for ChikVLP titer determination, similar to the approach utilized for quantifying influenza vaccine nanoparticles, encephalitic alphavirus VLPs [28], and HIV Env trimer [29]. The anion-exchanged resin derivatized with quaternary amine (TSKgel Q-STAT, 7 *μ*m, 4.6 mm ID × 10 cm, Tosoh, San Francisco, CA) retained the negatively charged particles present in the cell culture media, while allowing the neutral ChikVLPs to flow through and to be loaded directly onto the SEC column (SRT SEC-300, 5 *μ*m particle size, 300 Å pores, 7.8 × 150 mm, Sepax Technologies, Inc., Newark, DE) followed by FLR detection (Ex 278 nm/Em 340 nm).

The SEC column did not retain ChikVLPs, either due to the exclusion of these large particles (~70 nm) from the relatively small size pores of the SEC column, while retaining the smaller medium constituents and cell secretion products. Such tandem separation resulted in highly efficient purification of ChikVLP from all the charged and smaller size impurities. ChikVLP eluted as a standalone peak at the time corresponding to the chromatographic system voids volume, and the titer was determined by measuring its FLR peak area against the standard calibration curve.

### 4.4. GFP Fluorescence Measurements

200 *μ*L of culture was sampled daily from each vessel and spun down at 200 × g to isolate cells. 150 *μ*L of the supernatant was transferred to a 96-well black biochemical assay plate for fluorescence readouts; the remaining supernatant was discarded. The cell pellets were resuspended in PBS to reach a concentration of 10*e*6 cells/mL, and 150 *μ*L of the resuspension was transferred to a 96-well plate for fluorescence readouts using a Spark® multimode microplate reader (Tecan Group Ltd., Switzerland). The samples were excited at 480 nm, and a single point emission readout at 520 nm was detected and recorded for the cell and supernatant fluorescence levels.

### 4.5. Plasmid Labeling

The plasmid DNA (VRC8900) was labeled using a Cy5-LabelIT kit (Mirus Bio). Each single kit was used to label ~200 *μ*g plasmid. Two bulk labeling experiments were carried out and mixed together to form a single lot to supply the Cy5-pDNA for all studies presented here. Each 6 mL bulk labeling was as follows: 1.2 mg pDNA, 300 *μ*L LabelIT Solution, and 600 *μ*L Buffer A were mixed with sterile Milli-Q water to reach a total volume of 6 mL. The mixture was incubated for 1 hr at 37°C for labeling to occur, then purified via ethanol precipitation. The final Cy5-pDNA pellet was resuspended in sterile Milli-Q water with a target concentration of 5 mg/mL and measured on a Nanodrop spectrophotometer (ThermoFisher, Waltham, MA). A correction factor was applied to correct for the Cy5 260 nm absorbance to determine the actual pDNA concentration.

### 4.6. PEI-DNA Complexing Kinetics

The experiment was carried out in 20 mL of FreeStyle 293 media in 2 × 125 mL shake flasks without cells. Prior to starting the complexing reaction, microtubes were prepared with and without 50 units of DNase I (Sigma). pDNA was added at a 1 : 50 dilution to each flask to a target concentration of 20 *μ*g/mL and swirled to mix. The PEI was then added to only one flask at a 1 : 50 dilution to a target concentration of 40 *μ*g/mL and immediately placed in the shaker (using the same control conditions stated in Cell Culture: Transfection, Expression, and Sampling). At 4 min, 200 *μ*L was removed from each flask and 100 *μ*L was added to the (-) DNase vial, and the other 100 *μ*L was added to the (+) DNase vial. At 10 min, the same procedure was performed. The samples were incubated for 5 min at room temperature and then run on a 0.8% agarose gel (E-Gel: Invitrogen, Carlsbad, CA).

### 4.7. Cell-PEI/DNA Complex Binding Kinetics

An identical procedure was performed for all time points, where the reported time point is the point at which the sample was removed from the flask, post PEI addition as described in Cell Culture: Transfection, Expression, and Sampling. For each time point, a 100 *μ*L sample of cells was taken from each flask and transferred to a microtube then immediately centrifuged for 90 sec at 200 × g. The flasks were returned to the shaker. The supernatant was removed, and the cells were resuspended in 1 mL of chilled 1x PBS (containing 3% fetal bovine serum (FBS)) to reach a 10x cell dilution. The cells were then analyzed on a Guava easyCyte 8HT flow cytometer.

### 4.8. Cell Staining and Flow Cytometry

For VLP expression analysis, the cell staining procedure was the same for all samples. For each analysis, based on the daily cell count, 1*e*6 viable cells were taken from the culture, spun down at 200 × g at 4°C, then washed with 1 mL of chilled 1x PBS/3% FBS, spun down again, and resuspended in 500 *μ*L of chilled 1x PBS/3% FBS. The cells were then stained with an Alexa488 labeled ChikVLP mAb using a 1 : 100 dilution and kept on rotation at 2-8°C, then spun down and resuspended in chilled 1x PBS/3% FBS. Propidium iodide (viability dye) was then added to each sample, and the cells were transferred to a plate for flow cytometry analysis. All flow cytometry samples were run on a Guava easyCyte 8HT, and 20,000 events were recorded for each sample prior to applying gates. The forward and side light scatter was used to exclude debris and isolate singlet cells ([Supplementary-material supplementary-material-1]). The blue (488 nm) laser was used to excite the Alexa488-ChikVLP mAb and propidium iodide, and the respective fluorescence was captured using the green (525/30 nm) and red1 (690/50 nm) filter channels. The red (635 nm) laser was used to excite the Cy5-pDNA, and fluorescence staining was captured using the red2 (661/19 nm) filter channel. The pDNA fluorophore and ChikVLP fluorophore were chosen strategically based on separate excitation lasers to completely avoid fluorescence emission overlap in the signals or potential difficulties with compensation. Additionally, no fluorescence emission overflow was noted between the Alexa488/PI channels, so it was determined that compensation was not necessary.

## Supplementary Materials

Supplementary Materials Figure S1: gating strategy for all samples. Figure S2: (A) DNA agarose gel depicting the PEI-pDNA complexing kinetics and pDNA protection from enzyme degradation. (B) Flow cytometry results of PEI-DNA complex binding with cells at 3 hr for each transfection sample tested. Transfection samples are as follows: (i) (-) Cy5 20*e*6 cells/mL, (ii) 15*e*6 cells/mL, (iii) 20*e*6 cells/mL, and (iv) 25*e*6 cells/mL. The transfection efficiency (% TF) for each sample is listed above the graph. Figure S3: daily plasmid signal degradation tracking based on pDNA MFI. The plasmid signal was normalized by dividing the MFI for each sample by the daily background MFI signal in the nontransfected sample. Table S1: cell counts, viabilities, and specific productivities (Qp) for all transfection density (TFD) conditions tested. ^–^The day 4 Qp for the 15*e*6 samples is not reported due to titer assay error associated with complete cell lysis.
