# Supplementary material for: Comprehensive Flow Cytometry Analysis of PEI-Based Transfections for Virus-Like Particle Production
Source: Research (Wash D C). 2020 Mar 13;2020:1387402. doi: 10.34133/2020/1387402 (PMC7094759; doi:10.34133/2020/1387402)
Supplement: Supplementary Materials — Figure S1: gating strategy for all samples. Figure S2: (A) DNA agarose gel depicting the PEI-pDNA complexing kinetics and pDNA protection from enzyme degradation. (B) Flow cytometry results of PEI-DNA complex binding with cells at 3 hr for each transfection sample tested. Transfection samples are as follows: (i) (-) Cy5 20e6 cells/mL, (ii) 15e6 cells/mL, (iii) 20e6 cells/mL, and (iv) 25e6 cells/mL. The transfection efficiency (% TF) for each sample is listed above the graph. Figure S3: daily plasmid signal degradation tracking based on pDNA MFI. The plasmid signal was normalized by dividing the MFI for each sample by the daily background MFI signal in the nontransfected sample. Table S1: cell counts, viabilities, and specific productivities (Qp) for all transfection density (TFD) conditions tested. –The day 4 Qp for the 15e6 samples is not reported due to titer assay error associated with complete cell lysis. [file 1387402.f1.pdf]

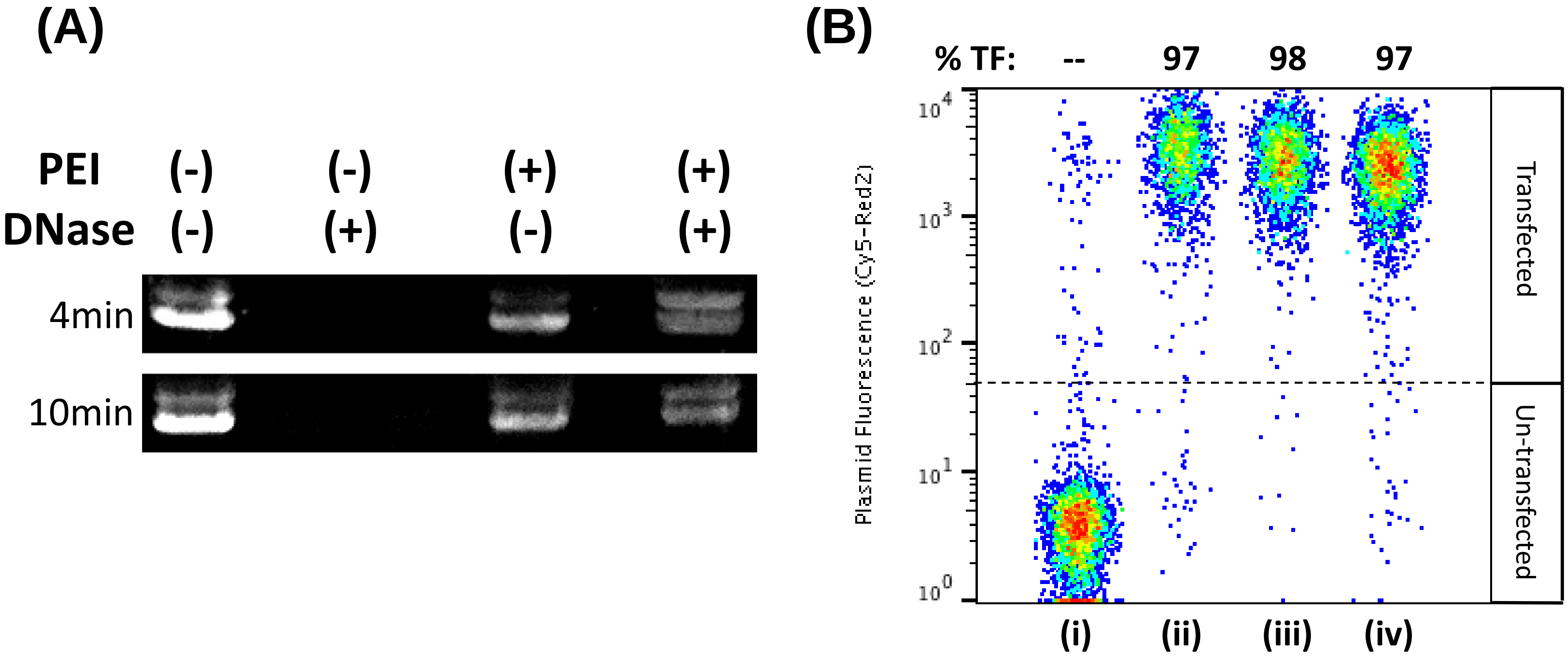

(A)
PEI (-) (-) (+) (+)
DNase (-) (+) (-) (+)
4min
10min
(B)
% TF: -- 97 98 97
Plasmid Fluorescence (Cy5-Red2)
10^4
10^3
10^2
10^1
10^0
Transfected
Un-transfected
(i) (ii) (iii) (iv)

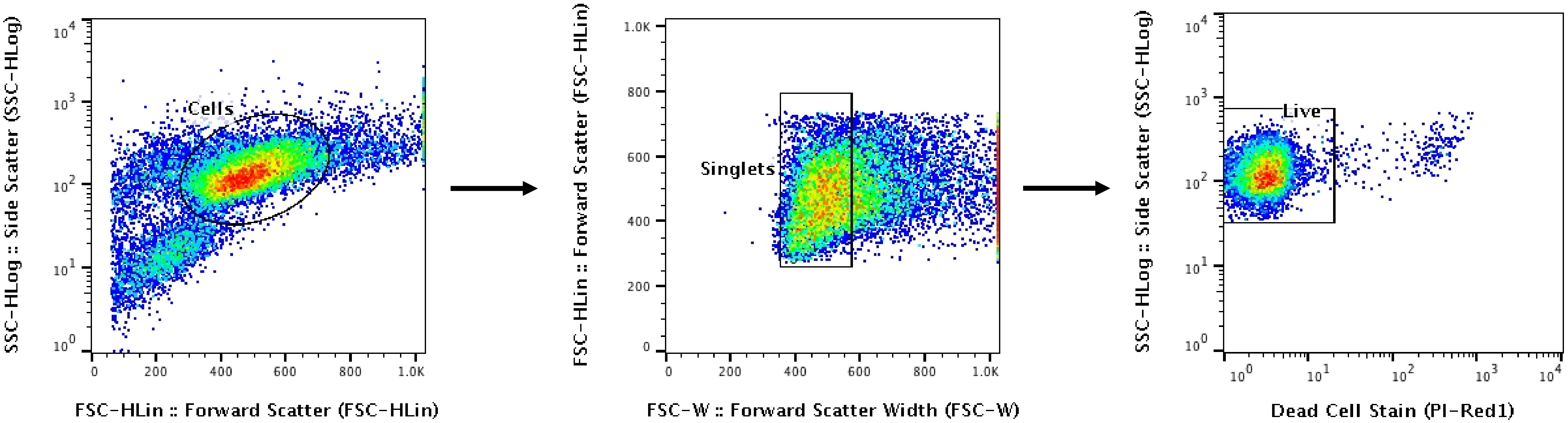
SSC-HLog :: Side Scatter (SSC-HLog)
Cells
FSC-HLin :: Forward Scatter (FSC-HLin)
FSC-HLin :: Forward Scatter (FSC-HLin)
Singlets
FSC-W :: Forward Scatter Width (FSC-W)
SSC-HLog :: Side Scatter (SSC-HLog)
Live
Dead Cell Stain (PI-Red1)

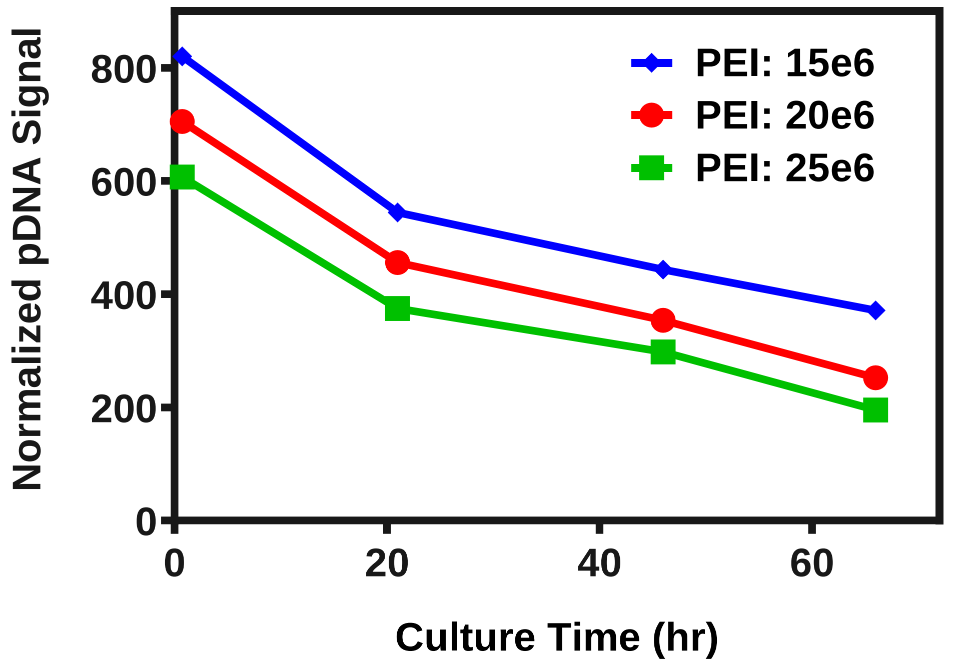
PEI: 15e6
PEI: 20e6
PEI: 25e6
Normalized pDNA Signal
0
200
400
600
800
0
20
40
60
Culture Time (hr)

Viable Cell Density (VCD) - [e6cells/mL]						
	(+) Cy5 Label			(-) Cy5 Label [avg. n=2]		
**Time (day)**	**TFD - 15e6**	**TFD - 20e6**	**TFD - 25e6**	**TFD - 15e6**	**TFD - 20e6**	**TFD - 25e6**
**0**	9.6	9.9	10.5	10.2	10.6	9.7
**1**	11.0	12.7	13.8	12.0	12.6	13.3
**2**	8.6	12.7	16.7	11.6	12.6	14.4
**3**	1.7	7.9	13.9	7.4	9.9	11.3
**4**	0.1	2.1	6.9	2.9	7.3	8.7

Viability [%]						
	(+) Cy5 Label			(-) Cy5 Label [avg. n=2]		
**Time (day)**	**TFD - 15e6**	**TFD - 20e6**	**TFD - 25e6**	**TFD - 15e6**	**TFD - 20e6**	**TFD - 25e6**
**0**	85	90	94	83	87	91
**1**	86	93	94	79	85	91
**2**	68	83	90	72	84	89
**3**	26	60	80	57	65	74
**4**	5	27	54	27	52	63

Specific Productivity (Qp) - [pg/cell/day]						
	(+) Cy5 Label			(-) Cy5 Label [avg. n=2]		
**Time (day)**	**TFD - 15e6**	**TFD - 20e6**	**TFD - 25e6**	**TFD - 15e6**	**TFD - 20e6**	**TFD - 25e6**
**0**	#N/A	#N/A	#N/A	#N/A	#N/A	#N/A
**1**	#N/A	#N/A	#N/A	0.3	0.4	0.4
**2**	0.5	0.5	0.4	-0.1	1.0	1.3
**3**	1.7	1.4	1.5	0.1	1.0	1.5
**4**	--	0.9	0.7	0	0.1	0.1
**Average**	1.1	0.9	0.9	0.1	0.6	0.8
